# Summer Diarrhoea

**Published:** 1913-08

**Authors:** C. O. Williams

**Affiliations:** West Point, Ga.


### SUMMER DIARRHOEA.

By C. O. Williams, M. D., West Point, Ga.

### Gastro-Enteritis, Sub-Acute Milk Infection.

This is a gastro-intestinal disorder, due to the toxins generated from the bacteria in milk; it usually occurs during the summer months when there is great humidity in the air; it occurs regularly each season being epidemic in large cities of the temperate zone.

Etiology. It is usually found among those fed bought milk, which milk contains millions of bacteria.

By sterilization it is possible to destroy the bacteria, but impossible to destroy all the toxines already formed, even at a temperature of 300 degrees F. This disease prevails to the extent to which other foods than breast milk is given to infants; even cows milk as ordinarily handled contains millions of bacteria which increases directly with the age of the milk and the height of temperature at which it is kept.

The weeds eaten by cows in their summer pastures are responsible for many cases of Gastro Intestinal Disorder; many of these weeds are poisonous and their juices pass into the milk.

In support of this theory "Hauser" gives statistics of infant mortality in a number of districts in his experience, classifying them by the soil and by the weeds which grow on certain soils; his table indicated a lower death rate on the granite and sand stone foundation.

Thus we learn by systematic eradication of weeds from the pasture and by keeping the cow, from which the baby's milk is obtained, away from such weeds and feeding from dry stall food and grass suitable for health and milk of the cow, which is a foster mother of the artificially fed infant, in this way we may vanish certain Gastro-Intestinal Infections in Infants.

Other predisposing factors are, age (under two years) feeble constitution unhygienic surroundings, malnutrition (improper food or improper feeding of proper food—as to quantity and intervals between feeding.

Bacteriology—Booker calls attention to three different forms of diarrhoea based on their clinical, anatomical, bacteriological features—

1—Dispeptic or non-inflammatory diarrhoea, in which the obligatory milk feces bacteria are found chiefly bacillus-coli-communis, and bacillus-lactis Aerogenes appearing in small numbers.

2—Streptococcus gastro interitis—in which there is an infection, and ulceration of the intestines with streptococci predominating.

3.—Bacilliary Gastro Enteritis—characterized by a general toxic condition and with less intestinal inflammation and the presence in the stools of several varieties of bacilli—the prteus vulgaris being the most common.

Pathology—Inflammatory lesions and ulcerations along the colon and the finding in the stools show numerous bacteria—epithelial cells, blood and particles of food.

Symptoms.—The symptoms are not so severe as those found in the acute form of milk infections; thus gastro-enteritis stands midway between acute indigestion and ileo-colitis;—vomiting and diarrhoea are the first and main symptoms in the beginning, and the only ones at times if treated early and properly; coated tongue, loss of appetite, temperature from 100 to 103 or often no temperature at first; green stools which are often characteristic and is an important symptom.

The irritation and tenesmus often cause the rectum to prolapse and from the constant discharge from the bowels we may find excoriation of the anus and buttock; the child is usually irritable, fretful, languid and depressed.

Diagnosis—The diagnosis can usually be made readily from the history and symptoms if given correctly by the mother or nurse with an inspection of the child undressed and carefully gone over and the character of the stools noted.

All the symptoms are much milder than in cholera-infantum; temperature lower, vomiting less, prostration not so marked.

Prognosis.—If it can be seen and handled early and without complications it is usually corrected.

Treatment.—Two points are to be considered.

1. Stop all milk for at least from 48 hours to a week or until symptoms are relieved.

2. Cleanse the stomach and bowels of all offending debris which may have caused this trouble. If vomiting persists, wash out stomach and then allow to remain at rest from 6 to 8 hours. For bowels—from 1-10 to 1-4 grain of calomel every two hours until effect is noted by frequency and character of stools changing for the better, then follow with castor oil which is mild, causing no griping, and is first cleansing and afterwards constipating; bismuth subgallate or subnitrate in large doses if diarrhoea persists; the sulphocarbolates of zinc, sodium, and calcium; a 1% solution of carbolic acid or beachwood creosote in same strength often gives good results. Enemas are often painful and cause the infant to fret and rebel against their uses and I think at times are of more harm and cause more worry and depression on the part of the patient than we derive good from them; however, the frequent mucous and muco-sanguinous stools accompanied with griping and strain and where the patient does not fret worse while giving enema, I think a warm salt solution or Glycothymoline or other mild antiseptics are cleansing, cooling and soothing to the lower bowels, for a short time at least, and often allows the patient to get quiet and off to sleep. For the persistent straining and tenesmus, which is often the most constant and distressing symptom of the little one, olive oil thrown in lower bowels, or asafoetida, or which I like better, a 50% emulsion of castor oil with 20 grains of argyrol to the ounce seems to quiet the bowels and relieve the congestion and irritation of the prolapse when found.

Feeding.—This is very important for the condition with which we are dealing and is very apt to depress the little patient even more than we expect, so strength and resisting power must be supported; the food should consist of light concentrated nutritious liquids given in small quantities and at short intervals. Barley water, rice water, oat meal water, whey, at first, and later these may be sweetened with granulated sugar from half to a teaspoonful to each feed, or half a teaspoonful of pure glycerine, or half grain of saccharine to each feed; also the yolk of one egg may be allowed to each feed as soon as the stomach will tolerate egg. Of course all babies fall off in weight when first taken off milk, so as soon as possible gradually put back to breast once or twice a day at first, and note character of stools, temperature, etc., lest the symptoms return; keep the patient out in fresh air, on the porch or in a park, send to the country, or to the coast, if patient is in position to spend the summer months away; for often a change in air and water will do much; have patient given frequent baths, beginning with the warm water and gradually cooling down. Stimulants are required in the majority of these cases and especially if of long duration—old brandy is probably best for general use or champagne if the stomach is very irritable; for an infant a year old about 1-2 ounce to 1 ounce and a half should be given in twenty-four hours. Stimulants should always be diluted with from six to eight parts water and given between feeds.

Drugs are of secondary consideration in this condition and are only given to relieve symptoms and are discontinued when this is effected—avoid opiates—rely on proper feeding, fresh air, and water.

